# Persistent *Listeria monocytogenes* Isolates from a Poultry-Processing Facility Form More Biofilm but Do Not Have a Greater Resistance to Disinfectants than Sporadic Strains

**DOI:** 10.3390/pathogens8040250

**Published:** 2019-11-20

**Authors:** Daniel Rodríguez-Campos, Cristina Rodríguez-Melcón, Carlos Alonso-Calleja, Rosa Capita

**Affiliations:** 1 Department of Food Hygiene and Technology, Veterinary Faculty, University of León, E-24071 León, Spain; drodrc01@estudiantes.unileon.es (D.R.-C.); crodm@unileon.es (C.R.-M.); carlos.alonso.calleja@unileon.es (C.A.-C.); 2 Institute of Food Science and Technology, University of León, E-24071 León, Spain

**Keywords:** *Listeria monocytogenes*, persistent strains, sporadic strains, biofilm formation, resistance to disinfectants

## Abstract

Some strains of *Listeria monocytogenes* can persist in food-processing environments, increasing the likelihood of the contamination of foodstuffs. To identify traits that contribute to bacterial persistence, a selection of persistent and sporadic *L. monocytogenes* isolates from a poultry-processing facility was investigated for biofilm-forming ability (crystal violet assay). The susceptibility of sessile cells to treatments (five minutes) with sodium hypochlorite having 10% active chlorine (SHY: 10,000 ppm, 25,000 ppm, and 50,000 ppm) and benzalkonium chloride (BZK: 2500 ppm, 10,000 ppm, and 25,000 ppm) was also studied. All isolates exhibited biofilm formation on polystyrene. Persistent strains showed larger (*p* < 0.001) biofilm formation (OD_580_ = 0.301 ± 0.097) than sporadic strains (OD_580_ = 0.188 ± 0.082). A greater susceptibility to disinfectants was observed for biofilms of persistent strains than for those of sporadic strains. The application of SHY reduced biofilms only for persistent strains. BZK increased OD_580_ in persistent strains (2500 ppm) and in sporadic strains (all concentrations). These results indicate that the use of BZK at the concentrations tested could represent a public health risk. Findings in this work suggest a link between persistence and biofilm formation, but do not support a relationship between persistence and the resistance of sessile cells to disinfectants.

## 1. Introduction

Listeriosis is a food-borne disease responsible for 23,000 infections yearly worldwide [1]. Its lethality rate may reach 30% [2]. In the United States, approximately 1600 cases of severe forms of human listeriosis occur each year (the incidence rate is 0.26 cases per 100,000 population), and about 260 of these die [3]. As a consequence of demographic changes, the country expects an increase in the prevalence of the disease, such that it will reach a figure of 0.32 cases per 100,000 inhabitants in 2030 [4]. Moreover, listeriosis is estimated to cause an annual loss of 8800 disease adjusted life years (DALYs) in the USA, of which the majority are premature deaths [5]. 

As for the European Union, in 2017 there were 2480 confirmed cases of invasive listeriosis (0.48 cases per 100,000 population). The lethality rate was 13.8% of the 1633 confirmed cases with a known outcome, the highest among all food-borne illnesses [6]. Moreover, over the last ten years, there has been, in the European Union, a substantial increase in the number of cases of human listeriosis, which reached 1381 (0.30 cases per 100,000 population) in 2008 [7]. Those suffering the infection most often and most severely are what are termed at-risk groups, such as the young, the old, the pregnant, and the immunocompromised [2].

Twenty species of *Listeria* have been described [8], but just three of them are pathogenic. Most cases of human listeriosis are caused by *Listeria monocytogenes*, although on rare occasions infections by *Listeria ivanovii* and *Listeria seeligeri* have been recorded [9]. With regard to *L. monocytogenes*, a total of 13 serotypes (1/2a, 1/2b, 1/2c, 3a, 3b, 3c, 4a, 4ab, 4b, 4c, 4d, 4e, and 7) have been described, although three of them (1/2a, 1/2b, and 4b) are responsible for the great majority of cases of human disease [10]. 

Persistent *L. monocytogenes* strains have been identified as major post-processing contaminants of foodstuffs, and numerous listeriosis outbreaks have been associated with the occurrence of persistent environmental contamination of processing plants [11]. Bacterial persistence is generally defined as the finding over the long term of genetically indistinguishable strains isolated from the same environment [12]. 

A relationship between biofilm formation and persistence has been suggested [13]. The sessile living state has been shown to be the preferred form of existence for many bacteria, and it is known that cells in biofilms show greater resistance to environmental challenges, such as desiccation, UV light, or sanitizers, than their planktonic counterparts in suspension [14,15]. This may lead to bacterial persistence in food-processing plants. However, the reasons for persistence remain poorly understood, and there is disagreement regarding the relationship between persistence and the ability to form biofilms [16].

Because long-term presence in food-processing plants poses health concerns and presents a risk factor for the economy, it is crucial to identify causes that encourage the persistence of *L. monocytogenes* in food-processing facilities. The aim of this research was to determine if there is a link between persistence and biofilm-forming capacity or resistance to disinfectants. To that end, a comparison was made of the biofilm-forming abilities of several persistent and sporadic *L. monocytogenes* strains isolated from a poultry-processing facility in north-western Spain. Additionally, the effects of different concentrations of sodium hypochlorite and benzalkonium chloride on the biofilms formed by these strains was evaluated. 

## 2. Results

### 2.1. Capacity of L. monocytogenes to Form Biofilm

**Figure 1:**
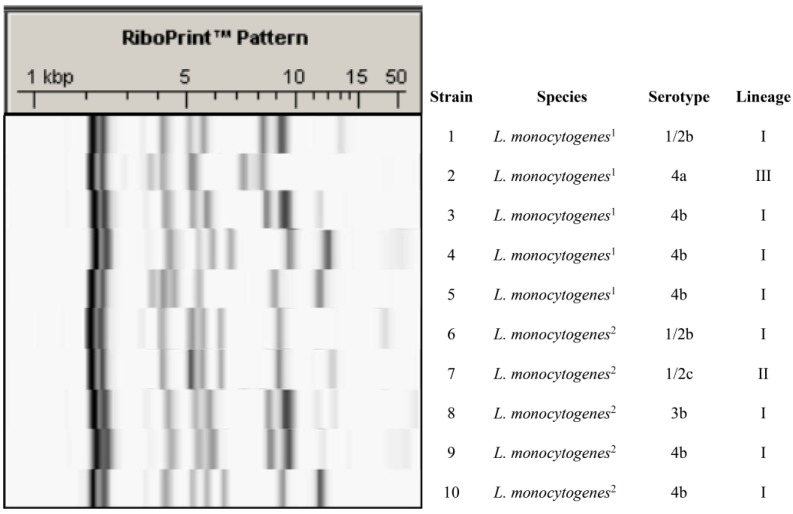
RiboPrint patterns of the 10 strains of *Listeria monocytogenes* tested. ^1^ persistent strains; ^2^ sporadic strains. RiboPrint patterns have been cropped from different gels.

The study included ten *L. monocytogenes* strains isolated from inert surfaces in a poultry-processing plant. The strains belonged to serotypes 1/2b (two strains), 1/2c (one strain), 3b (one strain), 4a (one strain), and 4b (five strains). Isolates were grouped into three phylogenetic lineages: I (strains 1, 3, 4, 5, 6, 8, 9, and 10), II (strain 7), and III (strain 2), as shown in Figure 1. 

All strains formed biofilms on the polystyrene surface under trial. On the basis of the cut-off OD_580_ (ODc) of 0.168, strains were classified as moderate (strain 2, serotype 4a) or weak producers of biofilms (remaining strains). 

The average value obtained for OD_580_ was 0.244 ± 0.106, with significant differences (*p* < 0.05) being seen between strains (Table 1). No differences were found in the abilities to form biofilms of the various groups of serotypes. The OD_580_ of strains of serotypes in group 1/2 (1, 6, and 7) showed a value of 0.230 ± 0.099, whilst the value for strains of serotypes in group 4 (strains 2, 3, 4, 5, 9, and 10) was 0.261 ± 0.107, and that for the strain of serotype 3b (strain 8) was 0.188 ± 0.107 (*p* > 0.05). 

By lineages, the OD_580_ of the strain in lineage III, at 0.361 ± 0.108, was a higher figure (*p* < 0.05) than the values observed for strains in lineages I (0.238 ± 0.096) and II (0.178 ± 0.104). When strains were grouped by persistence, values for OD_580_ of 0.301 ± 0.097 were observed for persistent strains, and of 0.188 ± 0.082 for sporadic strains (*p* < 0.001).

### 2.2. The Effect of Disinfectants on Biofilms

**Figure 2:**
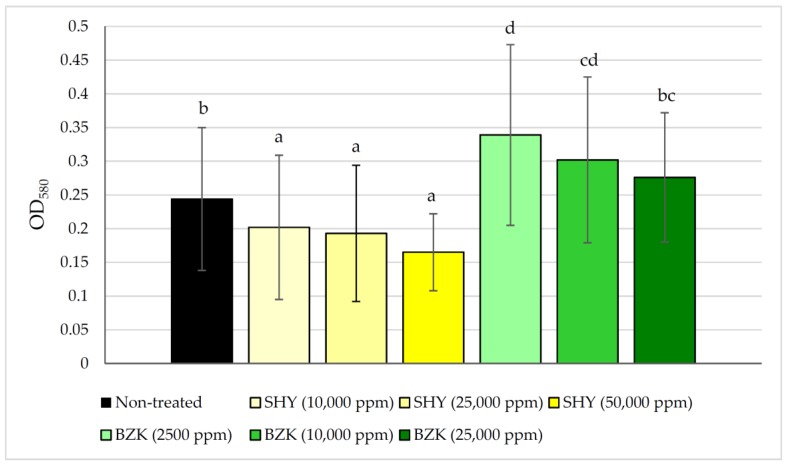
Biofilm formation by *Listeria* *monocytogenes* (average values for ten *Listeria monocytogenes* strains are shown) before and after treatment for five minutes with various concentrations of sodium hypochlorite (SHY) or benzalkonium chloride (BZK). Average values with no letters in common are significantly different (*p* < 0.05).

Figure 2 shows the values for OD_580_ averaged over ten strains that were found for untreated biofilms and for those treated with varying concentrations of SHY and BZK. Treatment with SHY reduced (*p* < 0.05) the amount of biofilm at all three concentrations assayed (10,000 ppm, 25,000 ppm, and 50,000 ppm), even if no major differences were observed relative to untreated biofilms. With regard to BZK, treatment with 25,000 ppm did not modify OD_580_ of biofilms (*p* > 0.05) relative to untreated biofilms. It must be noted that higher (*p* < 0.05) values for OD_580_ were observed for biofilms exposed to 2500 ppm and 10,000 ppm of BZK than for untreated biofilms. 

After treatment with SHY (10,000 ppm, 25,000 ppm or 50,000 ppm) no differences were observed between the OD_580_ of the various strains (Table 1). However, treatment with BZK (2500 ppm, 10,000 ppm and 25,000 ppm) caused considerable differences among the OD_580_ figures for different strains. Values were recorded that ran from 0.271 ± 0.131 to 0.493 ± 0.075 (treatment with BZK at 2500 ppm), from 0.229 ± 0.150 to 0.444 ± 0.106 (treatment with BZK at 10,000 ppm), and from 0.226 ± 0.095 to 0.388 ± 0.087 (treatment with BZK at 25,000 ppm). 

Table 2 gives the figures for OD_580_ of persistent and sporadic strains under all the conditions tested. Biofilms formed by persistent strains were more susceptible to disinfectants than those produced by sporadic strains. Thus, SHY reduced the biomass only of those biofilms formed by persistent strains, having no effect on sporadic strains. Treatment with BZK either increased (at 2500 ppm) or left unchanged (at 10,000 ppm and 25,000 ppm) the biofilms made by persistent strains. In the case of sporadic strains, all three concentrations of BZK increased the biomass of biofilms.

## 3. Discussion

### 3.1. Capacity of Strains of L. monocytogenes to Form Biofilm

Some of the serotypes of *L. monocytogenes* detected in samples taken from surfaces in the chicken-processing plant studied (1/2b, 1/2c, 3b, 4a, and 4b) are among those most often involved in human listeriosis. Specifically, strains of serotypes 1/2b and 4b, together with those of serotype 1/2a, are responsible for approximately 95% of instances of human illness worldwide [17]. 

The serotype with the highest percentage of strains was 4b, to which 50% of isolates were assigned (60% of persistent strains and 40% of sporadic strains). This result is similar to those noted in meat products by other authors [18,19,20,21], who observed that between 38.5% and 50% of strains isolated from foodstuffs belonged to serotype 4b. It should be pointed out that this serotype, besides being the type most often implicated in outbreaks of human listeriosis, appears to have greater potential for virulence than others, since it is the variety most often isolated from patients suffering from the most serious forms of the disease [17]. An important outbreak of listeriosis that occurred in Spain during August and September 2019 by chilled pork products, with more of 200 cases of human disease, three deaths, and seven miscarriages, has been linked to *L*. *monocytogenes* serotype 4b [22,23].

Strains in serotypes 1/2b, to which 20% of the isolates tested were ascribed, and 1/2c, with 10%, have often been detected in food, including meat products [17,24,25,26,27,28,29]. One strain (10% of the total) belonged to serotype 3a. This low percentage agrees with the findings of other researchers, who observed a low prevalence of strains of serotype 3a both in foodstuffs [17] and in clinical samples [29,30,31]. Serotype 4a, to which one strain (10%) was assigned, is not usually related to food-borne illness, even though it is often isolated from various environmental and food specimens, principally from ruminants and other non-primate mammals [32,33,34].

*L. monocytogenes* is divided into four evolutionary lineages (I, II, III, and IV), differing in their distribution and prevalence in the environment [35,36]. Strains belonging to lineage I (which includes serotypes 1/2b, 3b, 3c, and 4b) and II (1/2a, 1/2c, and 3a) are the most prevalent in human clinical isolates [32,33,34,37]. Specifically, cases forming part of outbreaks are related to lineage I, whilst sporadic cases are linked to lineage II [34]. Lineages III and IV are uncommon; they include serotypes 4a, 4c, and atypical cells of serotype 4b, and their presence is linked to animals [35,38]. In the work being reported here, lineage I included 80%, and lineage II 10%, of strains. The high percentage of strains in lineage I is a striking result, because it is unusual for them to appear in food-processing installations [33]. According to Orsi et al. [33], the apparent overrepresentation of lineage II among foods and food-processing environments may be due to an increased ability of strains in this lineage to grow and persist in these habitats.

There are several methodologies for determining biofilm physiology, structure, and composition. Among these, biofilm formation in polystyrene microtiter plates is certainly the most commonly used method [39,40]. The fact that all the strains assayed formed biofilms on polystyrene is a result coinciding with previous observations [14,15,41] and the findings of other authors [42,43]. This is a worrying discovery, because biofilms are a cause of contamination of food during processing; thus, representing a danger to public health.

Nonetheless, it must be pointed out that the capacity of strains to form biofilms was low. Nine of the ten isolates were only weak producers of biofilms and only one presented moderate formation of a biofilm. As in the present study, some researchers have also reported a predominance of strains of *L. monocytogenes* with only weak or moderate abilities to form biofilms, whether the isolates were of clinical or of food origin [42,44,45]. Nevertheless, it must be noted that there are discrepancies between studies, and an ability of *L. monocytogenes* to form moderate to strong biofilms has been reported by other authors [46,47,48]. The average OD_580_ observed (0.244 ± 0.106) coincides with the findings of Kadam et al. [43]. These authors found that after incubation in TSB at 37 °C, the average value for OD_595_ obtained lay approximately in the range 0.1 to 0.2. However, the values observed in the present study are lower than those in other research consulted, in which average values for OD_595_ as high as 0.55 to 0.93 [49] or 1.14 [50] were observed. The different wavelengths used to measure optical density may be partially responsible for variations between the results of different authors.

As reported in other studies [49,50,51,52,53,54,55,56], there was significant inter-strain variability in biofilm formation. Results in the work being reported here also coincide with the findings of Borucki et al. [49], who observed no significant relationship between the serotype of strains and their ability to form biofilms. Nevertheless, a greater production of biofilms by isolates of group 1/2 relative to other serotypes has frequently been reported [16,43,44,45,53,57]. It should be noted that the limited number of isolates used in the present research prevents strong conclusions from being obtained.

In the present work, it was noted that strains in lineage III formed the largest quantities of biofilm, while no differences were found between lineages I and II with respect to their biofilm-forming abilities. It has been reported that *L. monocytogenes* serotype 4a (Lineage III) has a strong ability to form biofilms on polystyrene [58]. Results from this work are also in agreement with findings from Cherifi et al. [59] and from Di Bonaventura et al. [60], who observed no differences between biofilms formed by lineages I and II. However, it must be pointed out that results obtained by different authors are very variable. In some reports [49,52,54,61,62], it was recorded that strains in lineage I produced less biofilm than those in lineage II. Along those lines, some authors have noted that the greater prevalence of strains from lineage II in food-processing contexts might be related to their stronger ability to form biofilms [36,44,57,61]. Nevertheless, other studies have showed larger biovolumes among strains from lineage I than among those in lineage II [51,63]. The culturing conditions (for instance, temperature or culture medium) and the variability between the strains used in the various studies might be the underlying cause for the divergences in reported data [61].

The persistence of *L. monocytogenes* in environments where food is processed poses a challenge for public health, not to mention a considerable financial impact. Hence, it is of importance to learn which characteristics favor the persistence of this bacterium in the installations and equipment of food industries. It was with the aim of determining whether a capacity to form biofilms and any resistance to disinfectants might influence the persistence of bacteria, that the present study was carried out on biofilms produced by ten strains of *L. monocytogenes* (five persistent and five sporadic isolates) before and after exposure to various concentrations of SHY and BZK. 

Since more biofilm was produced by persistent isolates than by transient sporadic isolates in the poultry-processing facility investigated, the present study suggests that biofilm formation may have contributed to persistence. Contradictory results have been found by other authors. Some studies showed that persistent strains of *L. monocytogenes* formed more biofilm than non-persistent strains [5,13,49,52,54,64,65]. Others observed no difference between persistent strains as opposed to sporadic strains [44,65,66,67,68]. Thus, the association between the ability of *L. monocytogenes* strains to form biofilms and their persistence in a food-processing plant environment is not clear. Differences in the results of the several studies may be related to inter-strain variability and to variations in methods applied, including sample size, temperature, pH, salt, nutrients, surface material, or growth medium [16]. It should be also noted that background microflora can affect the ability of *L. monocytogenes* to form a biofilm and to persist in *in vivo* systems [69,70].

### 3.2. The Effect of Disinfectants on Biofilms

The effects of SHY and BZK on biofilms of *L. monocytogenes* have been studied before [14,15]. However, the concentrations used (between 1750 ppm and 5250 ppm for SHY, and between 1.5 ppm and 19.5 ppm for BZK) were lower than in the present work. Here, the concentrations tested were between 10,000 ppm and 50,000 ppm for SHY, and between 2500 and 25,000 ppm for BZK. Furthermore, in earlier work, no comparisons were made between the biofilm-forming ability of persistent and transient-sporadic *L. monocytogenes* strains. 

The concentrations of disinfectants used in the current research (1000 to 5000 ppm of free chlorine and 2500 to 25,000 ppm of BZK) match the strengths generally used in the food industry, at 800 ppm to 2000 ppm of free chlorine for chlorine-based compounds, such as SHY, and 1000 ppm to 5000 ppm for quaternary ammonium compounds, such as BZK [71]. Since the disinfectant concentrations employed in this investigation proved of little effectiveness in eliminating biofilms of *L. monocytogenes*, especially in the case of BZK, it is suggested that in order to combat sessile cells of this bacterium efficaciously, the concentrations of these biocides normally used should be increased. However, additional research is warranted to substantiate this affirmation.

It was observed that contact between biofilms and BZK increased the amount of biomass (markedly so for concentrations of 2500 ppm). An enhanced ability to form a biofilm in the presence of BZK has previously been demonstrated for strains of *Escherichia coli* [72], methicillin-resistant *Staphylococcus aureus* (MRSA) [73], *Salmonella* [71,74], and *L. monocytogenes* [14]. In the present study, the biofilms were exposed to BZK for five minutes and the biocide was then eliminated, although it is probable that residual quantities remained in the wells of the microtiter plate. Between treatment with biocides and staining, 60 to 120 minutes elapsed. It is possible that during this period of time the strains exposed to BZK (in contact with residual amounts of the biocide) may have been able to synthesize biofilms to a greater extent than strains not exposed in this way. The explanation for this greater production of biofilm by the strains exposed to residual doses of BZK may have to do with the adaptational response of the bacteria [71,73]. Another possible explanation for the larger amount of biomass in biofilms observed after contact with BZK is that the disinfectant incremented the quantity of biofilm remaining adherent to the walls of the wells during the fixing and staining process. Nevertheless, further studies would be necessary to confirm these hypotheses. Our results demonstrate that the concentrations of BZK normally used not only are inefficacious in eliminating biofilms of *L. monocytogenes*, but also imply a risk to consumers when employed. This is because they might lead to the lingering of residual amounts of disinfectant, associated with an increase in the biomass in biofilms. Hence, independently of the causes of the greater quantity of biofilm after exposure to BZK, these results underline the need to review the concentrations of quaternary ammonium compounds used in food premises when the elimination of *L. monocytogenes* biofilms is intended. 

After treatment with SHY, all strains showed similar values for OD_580_. However, treatment with BZK gave rise to considerable variability between strains with respect of the biomass levels in biofilms. As the effect of BZK is strain-dependent, such results underline the need to screen a wide range of strains in trials intended to assess the effect of this disinfectant on biofilms of *L. monocytogenes*. 

The hypothesis that the persistence of *L. monocytogenes* is linked to resistance to disinfectants has been investigated in numerous studies [16]. However, an association between resistance to disinfectants and the persistence of the pathogen in different food processing environments has been demonstrated only in a few cases [11,52,75,76,77,78]. A clear link between persistence and increased disinfectant resistance was not recorded in some other studies [65,68,79,80,81]. To the best of our knowledge, this is the first report where the greater susceptibility to biocides of the biofilms by *L. monocytogenes* was observed in the case of the group of persistent strains (Table 2). These findings suggest that resistance to disinfectants in *L. monocytogenes* sessile cells is strain-dependent, but not associated with persistence. However, it should be noted that these results should be considered with caution because only ten bacterial strains were tested.

## 4. Materials and Methods

### 4.1. L. monocytogenes Strains

Five strains of persistent ribotypes and five transient sporadic strains were selected (Figure 1). Strains were isolated by environmental swabbing from various locations (both product contact and non-product contact surfaces) in a chicken-processing plant in the Province of León in north-western Spain at several times over a period of twelve months. Strains were considered persistent if they were isolated on two occasions at least six months apart and were genetically indistinguishable through ribotyping. Strains were considered sporadic when they were isolated only once over the twelve-month sampling period. 

Strains were stored at –50 °C in tryptone soya broth (TSB; Oxoid Ltd., Hampshire, England) supplemented with 20% (vol/vol) of glycerol. To make working cultures, the frozen cells were sub-cultured in TSB at 37 °C for 24 h. These cultures were then streaked onto tryptone soya agar (TSA; Oxoid) plates and incubated for 18 to 24 h at 37 °C.

### 4.2. Ribotyping and Serotyping

An automated RiboPrinter® microbial characterization system (DuPont Qualicon, Wilmington, Delaware, USA) was used in accordance with the manufacturer’s instructions to perform ribotyping [82]. Lysis of target cells to release cellular DNA, *Eco*RI digestion of the chromosomal DNA, separation of the resulting fragments by agarose gel electrophoresis, and hybridization with a chemo-luminescent-labelled DNA probe containing the *E. coli* ribosomal RNA operon were all carried out within eight hours. The numbers, positions, and relative intensities of rRNA operon-specific DNA fragments were estimated automatically by the RiboPrinter analysis software, and a digital record of 256 numerical values for each sample was produced. Linkage distances between patterns were estimated by means of Pearson’s correlation coefficient, and isolates were clustered by means of Ward’s method, using the Statistica® 8.0 software package (StatSoft Inc., Tulsa, Oklahoma, USA). Isolates with a linkage distance equal to or less than 0.05 (1-Pearson’s r) were considered indistinguishable (Figure 1). This threshold was established from a cluster analysis of ribotyping profiles for three *L. monocytogenes* collection strains in three different gels.

Serotyping was performed using the agglutination method performed with a Seiken *Listeria* antisera kit (Denka Seiken Co., Tokyo, Japan), in accordance with the manufacturer’s instructions. Strains were grouped into lineages on the basis of their serotype: lineage I, comprising serotypes 1/2b, 3b, 3c, and 4b, lineage II with serotypes 1/2a, 1/2c, and 3a, and lineages III and IV, comprising serotypes 4a and 4c [1,33]. 

### 4.3. Biofilm Determination

To quantify biofilms, a previously described procedure [83] was followed. Strains cultured on TSA were transferred to TSB and incubated for 18 h at 37 °C. Once this time had elapsed, the tubes held a concentration of approximately 10^9^ cfu/mL. Four decimal dilutions in TSB were performed to yield concentrations of 10^5^ cfu/mL, which were then used to inoculate the wells of polystyrene microtitre plates (Oy Growth Curves Ab Ltd., Finland). The wells were filled with 225 µL of TSB and 25 µL of bacterial culture, so that the final concentration in the well was 10^4^ cfu/mL. Negative controls were included, containing 250 µL of TSB. The microtiter plates were incubated at 37 °C for 24 h. 

To study biofilms in the absence of disinfectants, the content of the plate was poured off and the wells washed with 300 µL of sterilized distilled water. The bacteria that remained attached were fixed by adding 250 µL of methanol to each well for 15 minutes. The plates were then emptied, air dried, and stained for five minutes with 250 µL per well of an aqueous solution of 0.5% crystal violet. The wells were then emptied and washed by placing the plate under running water from the tap. The plates were air dried, and then the dye bound to the adherent cells was re-solubilized with 250 µL of 33% acetic acid (Sigma-Aldrich Co., St. Louis, Missouri USA) per well, the substance being allowed to work for one minute. Optical density at 580 nm (OD_580_) was determined in a Bioscreen C MBR (Oy Growth Curves Ab). The micro-well plates were agitated for one minute prior to the measurement of turbidity.

To investigate the effect of disinfectants on biofilms, the strains were incubated at 37 °C for 24 h in the wells. These were then emptied and refilled with 250 µL of an aqueous solution of the appropriate disinfectant. The substances used were sodium hypochlorite with 10% free chlorine (SHY; Sigma-Aldrich) and benzalkonium chloride (BZK; Sigma-Aldrich). SHY was used at concentrations of 10,000 ppm (1000 ppm of free chlorine), 25,000 ppm (2500 ppm of free chlorine), and 50,000 ppm (5000 ppm of free chlorine). BZK was used at 2500 ppm, 10,000 ppm, and 25,000 ppm. All the solutions were allowed to work at room temperature for five minutes. Thereafter, the wells were emptied and the procedure for staining and measuring OD_580_ cited above was performed. All experiments were replicated six times on separate days.

Strains were classified as a function of their capacity to form biofilms. The cut-off OD_580_ (ODc) was defined as three standard deviations above the mean OD of the negative controls. Strains were split into four categories: not biofilm producers, when OD_580_ ≤ ODc; weak biofilm producers, when ODc < OD_580_ ≤ (2ODc); moderate biofilm producers, when (2ODc) < OD_580_ ≤ (4ODc); or strong biofilm producers, when (4ODc) < OD_580_ [81]. 

Control strains that were strong and weak formers of biofilm from the collection of strains of the University of León, Spain, were included in each experiment. Differences in the extent of biofilm formation were examined by analysis of variance (ANOVA) techniques. Mean separations were obtained using Duncan’s multiple range test. Significance was determined at the *p* < 0.05 level. All data processing in this study was carried out using the Statistica® 8.0 software package (StatSoft Ltd., Tulsa, Oklahoma, USA).

## 5. Conclusions

All the strains of *L. monocytogenes* tested were able to form biofilm on polystyrene surfaces, a worrying fact in the context of food safety, in view of the extensive use made of plastics in food-processing installations. The capacity of isolates to form biofilms was strain-dependent, but not associated with the serotype. Furthermore, significant differences in biofilm formation between persistent and non-persistent strains of *L. monocytogenes* were observed, with persistent strains showing the strongest biofilm-forming ability. Treatment with SHY brought about significant reductions only in the biofilms of persistent strains. BZK increased the amount of biofilm remaining after the disinfection process, more markedly so with sporadic strains. This fact makes plain the potential risk arising from the use of this substance at the concentrations studied. Results in this study suggest that *L. monocytogenes* may persist thanks to its biofilm-forming capacity, which promotes its survival in food-processing facilities. In contrast, sanitizer tolerance does not appear to affect the persistence of strains. 

## Figures and Tables

**Table 1:** Biofilm quantities (crystal violet assay; OD_580_) of ten *Listeria monocytogenes* isolates before (control) and after exposure to sodium hypochlorite or benzalkonium chloride at various concentrations.

Strain (Serotype)	Treatment (Five Minutes)						
	CONTROL	Sodium Hypochlorite (10% of Active Chlorine)			Benzalkonium Chloride		
	Without Treatment	10,000 ppm	25,000 ppm	50,000 ppm	2500 ppm	10,000 ppm	25,000 ppm
**1 (1/2b)**	0.318 ± 0.053 ^abc^_cd_	0.261 ± 0.191 ^ab^_a_	0.237 ± 0.187 ^ab^_a_	0.207 ± 0.049 ^a^_a_	0.493 ± 0.075 ^d^_d_	0.436 ± 0.072 ^cd^_b_	0.372 ± 0.090 ^bcd^_bc_
**2 (4a)**	0.361 ± 0.108 ^abc^_d_	0.284 ± 0.200 ^abc^_a_	0.218 ± 0.194 ^ab^_a_	0.201 ± 0.074 ^a^_a_	0.457 ± 0.146 ^c^_cd_	0.444 ± 0.106 ^c^_b_	0.388 ± 0.087 ^bc^_c_
**3 (4b)**	0.244 ± 0.108 ^ab^_abcd_	0.166 ± 0.044 ^ab^_a_	0.184 ± 0.044 ^ab^_a_	0.148 ± 0.061 ^a^_a_	0.273 ± 0.132 ^b^_a_	0.258 ± 0.085 ^ab^_a_	0.233 ± 0.057 ^ab^_a_
**4 (4b)**	0.308 ± 0.103 ^b^_bcd_	0.229 ± 0.092 ^ab^_a_	0.225 ± 0.048 ^ab^_a_	0.172 ± 0.054 ^a^_a_	0.317 ± 0.119 ^b^_ab_	0.319 ± 0.121 ^b^_ab_	0.294 ± 0.092 ^b^_abc_
**5 (4b)**	0.274 ± 0.092 ^bc^_abcd_	0.181 ± 0.087 ^ab^_a_	0.163 ± 0.072 ^ab^_a_	0.153 ± 0.050 ^a^_a_	0.306 ± 0.126 ^c^_ab_	0.263 ± 0.099 ^abc^_a_	0.276 ± 0.065 ^bc^_ab_
**6 (1/2b)**	0.193 ± 0.075 ^ab^_ab_	0.161 ± 0.035 ^a^_a_	0.151 ± 0.034 ^a^_a_	0.146 ± 0.057 ^a^_a_	0.271 ± 0.131 ^b^_a_	0.247 ± 0.094 ^ab^_a_	0.226 ± 0.095 ^ab^_a_
**7 (1/2c)**	0.178 ± 0.104 ^abc^_a_	0.156 ± 0.061 ^ab^_a_	0.185 ± 0.095 ^abc^_a_	0.147 ± 0.049 ^a^_a_	0.300 ± 0.081 ^d^_ab_	0.278 ± 0.090 ^cd^_a_	0.250 ± 0.051 ^bcd^_a_
**8 (3b)**	0.188 ± 0.107 ^ab^_a_	0.211 ± 0.074 ^ab^_a_	0.218 ± 0.094 ^ab^_a_	0.141 ± 0.046 ^a^_a_	0.347 ± 0.112 ^c^_abc_	0.284 ± 0.105 ^bc^_a_	0.257 ± 0.078 ^abc^_a_
**9 (4b)**	0.176 ± 0.069 ^a^_a_	0.188 ± 0.079 ^a^_a_	0.157 ± 0.045 ^a^_a_	0.186 ± 0.077 ^a^_a_	0.288 ± 0.141 ^a^_a_	0.229 ± 0.150 ^a^_a_	0.231 ± 0.119 ^a^_a_
**10 (4b)**	0.205 ± 0.072 ^a^_abc_	0.181 ± 0.060 ^a^_a_	0.187 ± 0.083 ^a^_a_	0.150 ± 0.025 ^a^_a_	0.343 ± 0.134 ^b^_abc_	0.266 ± 0.134 ^ab^_a_	0.232 ± 0.081 ^ab^_a_

Average values (*n* = 6) in the same row without any letter in common (superscript) are significatively different (*p* < 0.05). Average values in the same column without any letter in common (subscript) are significatively different (*p* < 0.05).

**Table 2:** Biofilm quantities (crystal violet assay; OD_580_) of five persistent and five sporadic *Listeria monocytogenes* isolates before (control) and after exposure to sodium hypochlorite or benzalkonium chloride at different concentrations.

Group of Strains	Treatment (Five Minutes)						
	CONTROL	Sodium Hypochlorite (10% of Active Chlorine)			Benzalkonium Chloride		
	Without Treatment	10,000 ppm	25,000 ppm	50,000 ppm	2500 ppm	10,000 ppm	25,000 ppm
**Persistent**	0.301 ± 0.097 ^b^_a_	0.224 ± 0.136 ^a^_a_	0.206 ± 0.122 ^a^_a_	0.177 ± 0.059 ^a^_a_	0.369 ± 0.145 ^c^_a_	0.344 ± 0.123 ^bc^_a_	0.313 ± 0.095 ^bc^_a_
**Sporadic**	0.188 ± 0.082 ^a^_b_	0.180 ± 0.062 ^a^_a_	0.180 ± 0.073 ^a^_a_	0.154 ± 0.052 ^a^_a_	0.310 ± 0.117 ^c^_a_	0.260 ± 0.110 ^b^_b_	0.239 ± 0.082 ^b^_b_

Average values (*n* = 30) in the same row without any letter in common (superscript) are significantly different (*p* < 0.05). Data in the same column without any letter in common (subscript) are significantly different (*p* < 0.01). Five persistent strains of serotypes 1/2b, 4a, and 4b, and five sporadic strains of serotypes 1/2b, 1/2c, 3b, and 4b, were studied. For additional interpretation, see Figure 1.
